# An Essay on the Treatment of Spina Bifida (*Hydrorachitis*), by Injections of Iodine

**Published:** 1859-09

**Authors:** Daniel Brainard

**Affiliations:** Prof. of Surgery in Rush Medical College, Surgeon of the Chicago City Hospital, etc.


THE CHICAGO

MEDICAL JOURNAL.

VOL. II. SEPTEMBER, 1859. No. 9.

### ORIGINAL COMMUNICATIONS.

ARTICLE I.

### AN ESSAY ON THE TREATMENT OF SPINA BIFIDA (*HYDRORACHITIS*) BY INJECTIONS OF IODINE.

BY DANIEL BRAINARD, M. D.,
Prof. of Surgery in Rush Medical College, Surgeon of the Chicago City Hospital, etc.

This disease or malformation is generally regarded in this country as beyond the reach of remedy. I have often had occasion to verify the truth of this statement; and if we look into surgical books of authority but a few years since, we shall find that this view is only the perpetuation of an error then quite general everywhere. Thus, in the latest editions of Mr. Samuel Cooper, we find the following opinion concerning it: "The present affection is one of a most incurable nature; for, with the exception of one case by Morgagni, a second recorded by Kellman, and two or three more recently published by Sir Astley Cooper, there is not, I believe, in all the records of medicine or surgery any cases which either got well of themselves, or were benefited by any mode of treatment." Vidal de Cassis, Ollivier, an excellent authority on this subject, Sabbatier, Itard and others, advise against any operation. I hope to prove herein that such is not by any means generally the nature of this affection.

Another error generally prevalent among physicians is the opinion that spina bifida is of rare occurrence. Chaussier (*Dict.*

*de Medicine*, vol. xvi.) states that in the Foundling Hospital in Paris it is the malformation of most frequent occurrence, ranking before club-foot and hare-lip. This statement of Chaussier, although correct in itself, is not so in a general sense.

There are particular years in which this malformation is of frequent occurrence. They are those which follow periods of distress, as after epidemic diseases have prevailed extensively, etc. During the year 1849, which succeeded the cholera year, I met with four cases of spina bifida in new born children; while, from 1853 to 1858, in a much larger field of observation, I have only met with one.

Since 1836, I have treated one hundred and fifty cases of hare-lip, and near one hundred and thirty cases of club-foot. During the same period, I have only met with eleven cases of spina bifida of all kinds, of which five only were subjected to treatment. Making allowance for the fact that parents and physicians generally consider this latter as incurable, and therefore do not consult a surgeon, while the former are usually presented at an early age, it still appears that hare-lip and club-foot are each more frequent than spina bifida in the proportion of, at least, ten to one.

Spina bifida presents itself in form of tumor, situated along the course of the spinal column, or upon the occiput. It may vary in size from that of a child's head to that of a small nut, but it is most frequently found of the size of a common apple. It is circular or oval, sometimes pediculated, lobulated or double.

The most frequent situation is the lumbar region, or at its junction with the dorsal, next the sacral. It is, however, sometimes found upon the back part of the head. I once met with a case of the last named variety in a young girl, aged about six years. The tumor was situated at the occipital protuberance, and was of the size and shape of a pear, with a small end attached to the head. I regret that I was unable to procure a figure of this rare tumor. Those of the head or neck are esteemed the most dangerous, but this is doubtful.

The tumor is generally covered with healthy skin; the exceptions are where cicatrices exist, in some cases indicating a progress toward cure, where ulcerations exist, with or without appearance of cicatrices, when the covering at the centre is composed only of the membranes of the cord. This is seen in tumors of large size, and such often burst during childbirth.

The sac is lined by the arachnoid membrane and the dura mater. These are often thickened and indurated, and not endowed with the irritability of the normal arachnoid. The change which takes place is like that which is found in the tunica vaginalis in cases of hydrocele of long standing. The tumor is more or less soft and fluctuating according to the state of the circulation, *is hard during sleep*, translucent, as may be easily seen by placing the small end of the stethoscope against it and looking in while a light is applied to the other side. Where the walls are lax, the finger may be inserted between the transverse processes of one or more of the vertebræ. The spinous process is wanting.

The fluid contained in the tumor is generally clear, slightly yellow, and when allowed to stand, a fine coagulum of fibrine is seen to have formed in it. Flakes of albumen are also formed in it by the application of heat. Sometimes the cord itself is protruded through the opening, constituting a species of "hernia" (Olivier, *Dict. de Medicine*), and more frequently the nerves are found adherent to the walls of the sac. Indeed, Hewitt considers this to be the usual arrangement, and says, that in twenty preparations of spina bifida, he found but one in which it was otherwise (*Med. Gazette* for 1844).

The fluid of the sac can generally be pressed in part into the spine. Sometimes, however, the opening is closed. I have met with one case in which none of the fluid could be pressed into the spine, and in which pressure had no effect on the brain, and Velpeau regards the disease as sometimes of the nature of a serous cyst.

Among the varieties which this malformation presents are mentioned instances where it was situated on the side of the spine, and there is one instance on record where it was in the pelvis, and the bodies of the vertebræ divided. Among the complications are to be reckoned hydrocephalus, imperfection of the cord at the point where the disease is situated, club-foot, various deviations of the lower extremities and paralysis.

Several writers state that hydrocephalus is generally present, among whom may be mentioned Chelius, Vidal and others.

Chaussier, however, who founds his opinion on an examination of 132 cases, states that in the majority the disease is simple, and that any complication is to be regarded as an exception.

### NATURE AND SEAT OF THE DISEASE.

*Hydrorachitis* is undoubtedly to be regarded as an unnatural increase of the *cerebro-spinal* fluid; at least, of that part of it situated within the spine. Of this the following account is given by Todd and Bowman: "This fluid fills the sub-arachnoid space during life. Its quantity, which varies between two and ten ounces, is in the inverse ratio to the bulk of the brain and spinal cord." "The cerebro-spinal fluid is most probably secreted by the pia mater, since it is found wherever that membrane and sufficient space exist. The ventricles of the brain contain a secretion of very similar, if not identical, characters, which Magendie describes as communicating with that of the sub-arachnoid space through an orifice at the inferior extremity of the fourth ventricle. This, however, is extremely doubtful, as the fluid is enclosed by a membrane which lines their cavities." (*Physiological Anat.*, p. 231.)

We have then in the healthy state a fluid beneath the spinal arachnoid, a fluid in the ventricles, and a very slight quantity between the arachnoid and the dura mater.

In disease, an increase of that of the spine constitutes *hydrorachitis;* an increase of that of the ventricles, *hydrocephalus.* The cavities of the sub-arachnoid space do certainly communicate with the ventricles in some rare cases of disease, and then the two diseases co-exist, and with communication. The arachnoid is also often broken in disease so as to allow the fluid to pass into the space between it and the dura mater. This statement of Todd and Bowman corresponds exactly with pathological reports of dissections, and with physiological facts, for the separate functions of the cord and the brain would lead us to expect a certain degree of independence in the circulation of each.

Where the fluid of spina bifida is situated in the sac of the arachnoid membrane, it can only communicate with that in the lateral ventricles by a rupture of the membrane. Ollivier states that the fluid is often found beneath the arachnoid, and between it and the pia mater; and Meckel says this is its usual seat.

*Treatment.*—There are, as I have stated, many surgeons, and those among the most judicious, who oppose all surgical interference in this affection. I have already quoted Vidal and others to this effect, and new names might be added. I am happy to say, however, that in recent years this view is becoming less frequent than it was formerly.

The propriety of any treatment depends upon its results. After a review of what has been done, we shall be better able to judge of the value of the different operations proposed.

These are:

1. The seton, said to have been proposed by Dessault and Mathey, has been used several times, but always without success.

2. The ligature, proposed by Forestius and recommended by B. Bell, Reynard and L. de Thimecour.

3. Excision and closure of the opening with the suture, proposed by Dubourg.

4. Repeated puncture.

5. Pressure, proposed by Abernethy.

6. Injection of solution of iodine, proposed and performed by the author; performed also by Chassaignac, Velpeau and Nelaton.

7. Caustic issues near the tumor were proposed by Richter.

TABLE OF OPERATIONS FOR SPINA BIFIDA.

*Puncture.*

| | |
|---|---|
| 1, Laboune, 5 punctures, . . . . . . . | cure. |
| 1, Probart, not stated, . . . . . . . | " |
| 2, Rozetti, small punctures, . . . . . . . | " |
| 1, Wardrop, several punctures, . . . . . . | death. |
| 1, Hewitt, . . . . . . . . . . | " |
| 1, Velpeau, 4 punctures with lancet, . . . . | " |
| 1, Abernethy, 10 punctures, . . . . . . . | " |

| | |
|---|---|
| 1, Otto (died of hydrocephalus), . . . . . | cure. |
| 1, Hoffman, . . . . . . . . | " |
| 1, Beaunier, . . . . . . . . | " |
| 1, Pooley, . . . . . . . . | death. |
| 1, Buggiare, 2 punctures after 1 rupture, . . | cure. |
| 1, Repeated by same, . . . . . . | " |
| 2, Dumville, . . . . . . . | 1 cure, 1 death. |
| 1, Stafford, . . . . . . . . | " |
| 1, Name not given (*Med. Chir. Review*, for July, 1837, p. 228), . . . . . . . . | " |
| 1, Stevens, of N. Y., with iris knife, puncture kept open, | cure. |
| 2, Cooper, . . . . . . . . | " |
| 1, Each, on occiput, . . . . . . . | death. |
| 1, Cooper, . . . . . . . . | " |
| 1, Robert, . . . . . . . . . | cure. |
| 1, Rosetti, . . . . . . . . | " |
| 1, Skinner, 70 operations, . . . . . | death. |
| 1, Dupuytren, result not known. | |
| 1, Philippe Boyer, . . . . . . . | " |
| 1, Robert (de Beaujon), . . . . . . . | cure. |
| 1, Hawthorn, puncture and pressure, . . . . | palliation. |
| 1, Tatum, from tetanus, . . . . . . . | death. |
| 1, Ogle, . . . . . . . . . | " |
| 1, Sir Philip Crampton, 22 punctures, . . . . | cure. |
| 1, Hutchinson, . . . . . . . . | death. |
| 1, Barnes, puncture and rupture, . . . . | " |

*Excision.*

| | |
|---|---|
| 2, Trowbridge, after constriction, . . . . . | cure. |
| 1, Sherwood, . . . . . . . . | " |
| 2, Dubourg, . . . . . . . . . | " |
| 1, Tavignot, . . . . . . . . . | death. |
| 1, Roux, . . . . . . . . . | " |
| 1, Nott, . . . . . . . . . . | cure. |
| 1, Mott (probably not connected with spine), . . | " |
| 1, Cowan, result not known. | |
| 1, Hilton, . . . . . . . . . . | death. |
| 1, Childs, . . . . . . . . . | " |

*Pressure.*

| | |
|---|---|
| 1, Abernethy, . . . . . . . . . | death. |
| 1, Cooper, . . . . . . . . . | palliation. |
| 1, Stueber, . . . . . . . . . | cure. |
| 1, Beaunier, . . . . . . . . . | " |
| 1, Recamier, . . . . . . . . . | " |

*Ligature.*

| | |
|---|---|
| 1, Heister, . . . . . . . . . | death. |
| 1, Reynard, . . . . . . . . . | cure. |
| 1, L. de Thimecour, . . . . . . . | " |
| 2, Trowbridge, failure several times, . . . . | " |
| Ligature applied a short time, . . | " |
| 1, Peige, ligature and excision, . . . . . . | " |
| 1, Dubois, with pins, . . . . . . . | death. |
| 1, Erichsen, . . . . . . . . . | " |

Cases of recovery after rupture: Serris, Ulhoorn, Nance, Stafford,—4.

*A resume of the above cases gives the following result:*

| | Cure. | Death. | Unknown. | Palliated. | Total. |
|---|---|---|---|---|---|
| Puncture, . . . . | 17 | 16 | 1 | 1 | 35 |
| Excision, . . . . . | 7 | 3 | 1 | — | 11 |
| Ligature, . . . . . | 5 | 3 | — | — | 8 |
| Pressure, . . . . . | 3 | 1 | — | 1 | 5 |
| | 32 | 23 | 2 | 2 | 59 |

Chelius says that repeated punctures nearly always result unfavorably (*System of Surgery*, by South, vol. 3, p. 187).

Brechet, who tried puncture on a large scale, found it always to fail.

M. Pacoud operated several cases and always failed.

M. Bajard did the same with the same results.

Besides Sulzman, Trompei, Porter.

M. Lyon states that he has punctured seven or eight cases of spina bifida without benefit.

Indeed, I do not regard this table as of much practical value. Most frequently cases are reported so imperfectly as to be of little utility. It is for this reason that I have not sought to make it more extensive.

There are, however, two practical inferences which may be drawn from these cases. They are:

1st. The success of puncture is proportioned to the smallness of the instrument.

2nd. That when the fluid became turbid it indicated a favorable progress towards cure.

*Injections of Iodine.*—The cases operated on by injections of iodine, up to the present time, are ten in number; of these, five belong to the author, two were treated under my direction by Dr. S. G. Crawford, formerly of this city, one was treated by Chassaignac, one by Velpeau, and one by Nelaton.

I have no report in detail of the cases treated in Paris. Such as have been published will be added after the detail of the cases treated under my own observations.

CASE I.—The subject was a girl thirteen years of age. The tumor, situated at the top of the sacrum, was nine inches in circumference and three inches in height. Its surface and the skin adjoining presented numerous cicatrices, marks apparently of former ulcerations. The child was partially paralytic in the lower extremities, idiotic, and passed the urine and fæces involuntarily. The head was small and the bones perfectly formed.

The first injection was performed on the 2nd December, 1847. A puncture was made with the point of the lancet half an inch distant from the base of the tumor, and a small-sized exploring trochar carried thence into the sac. Through the canula, a solution of half a grain of iodine, with one grain of iodide of potassium in one ounce of distilled water, was injected. The canula was immediately withdrawn, and a compress and bandage applied so as to prevent the escape of the liquid.

The injection produced a sharp pain which soon subsided. Redness, heat, and tenderness of the tumor followed, for which a cathartic was administered, and evaporating lotions applied to the part. Compression was resorted to as the heat and tension subsided, and, December 27th, the tumor was about half its former size.

At this time, a second injection was resorted to of half the strength of the first. This produced little inflammation. The compression was continued. January 15, 1848, the fluid was so far absorbed as to render it easy to press most of it within the spine, and a common spring truss for hernia was applied, the pad upon the opening.

The case then passed from under my observation, but fell under the care of Dr. Huber, who had at that time charge of the county poor at Chicago. The following is his account of the case and his treatment:

"I injected the tumor thirteen times, viz., May 3, 10, 20, June 15, 22, July 14, August 10, 15, 25, September 5, 16, 26, and October 20, 1848. The injection for the first four times was of the strength of four grains of iodine and sixteen grains iodide of potassium to the ounce of distilled water, beginning with one and a half and increasing to three ounces at the fourth injection.

"The sac was then much contracted, and I therefore doubled the strength of the solution and injected but half an ounce. After the first two operations the child had some slight febrile symptoms, but not since. I consider the cure complete. She has improved in the use of her lower extremities, being now able to walk across the room."

This case was published in the *Chicago Medical Journal* for 1849, p. 495.

Two years after the operation the child remained cured, and much improved in every respect.

Case II.—This case occurred to me, April 12, 1849. The tumor was of the size of a closed fist, and had been ruptured during labor.

By the application of artificial heat, the first ill effects of the rupture were dissipated. A reddish serum was discharged from the sac and canal, which, on the 17th, became copious and offensive. Injected a solution of iodine, 4 grs. iodide potassium, 12 grs., to the ounce of distilled water. The solution escaped as fast as injected.

30*th.* Repeated injection; discharge purulent.

*May 2d.* Injected with solution of sulphate of copper, and applied compression over the opening. The sac was contracted down to a hard tubercle, with a small opening in the centre.

The injection was repeated on the 4th and 6th, and the child seemed well in every respect.

*May 12th.* Opening quite closed; *head noticed to be enlarging; bones separated.* This continued till June 1, when the child died in a paroxysm of convulsion.

This case resulted fatally from closing the fistulous opening in the skin too soon, but it illustrates, in a striking manner, the beneficial effect of injections into the spinal canal, and the little danger to be apprehended from their use. The child lived over six weeks.

I was not aware at the time of the danger of making compression over such an opening, but held, in common with the whole profession, the erroneous opinion that the danger in cases of inflammation of the meninges of the cord and brain was greatest when an opening existed.

This is an error which has recently been pointed out by Dr. Thompson, of Columbus, Ohio. Far from closing such an opening, the sac should be punctured if acute inflammation results from treatment, and the liquid drawn off.

Published in the *Chicago Medical Journal* for 1850.

CASE III.—*Spina Bifida, accompanied by Hydrocephalus.*

*Feb.* 12, 1850. A female child, three months old, born at seven months, and very feeble, was presented, affected with spina bifida and hydrocephalus. The tumor occupied the lumbar region, was three and a half inches in length, two and a half in breadth, and one and a half in elevation.

In the usual manner I injected it, using iodine ½ grain, iodide potassium 1½ grains, in 1 drachm of distilled water, without drawing off any fluid.

This was repeated but once. In the interval, ⅙ grain of tannic acid, in ½ drachm of distilled water, was injected at intervals of eight days.

After the last injection, which was of iodine, twitching of the members occurred, to such a degree as to induce me to draw off the liquid and replace it in part with distilled water. This gave immediate relief. The child took the breast and remained well for eighteen hours, when it took convulsions and died.

The opening into the spinal canal was perfectly closed. The sac contained one drachm of clear water, without any effusion of lymph or unnatural vascularity. There was no sign of irritation about the tumor to account for death. The head was large, measuring 18 inches in circumference. An examination of it was not allowed.

The tannic acid was twice substituted for the iodine at the suggestion of a medical friend, under the supposition that its astringency would be more effective in preventing effusion. It was found, however, to produce more irritation with less tendency to absorption afterward, and was therefore discontinued.

Although death occurred in this case suddenly, there is no reason to attribute it in any degree to the spina bifida, or to the treatment employed; it was the result of other causes, probably the disease of the head. The effect of four injections in as many weeks in closing the opening into the spine, without giving rise to suppuration or considerable inflammation, must be regarded as highly favorable results of the treatment.

Case IV.—*Sept. 20th*, 1849. Saw a female child, aged four days old, affected with spina bifida and hydrocephalus. The tumor was about three inches in length and half as broad. There seemed to be a division of the bodies of some of the vertebræ. The covering of the tumor was semi-transparent, and seemed to be composed of the membranes of the cord and cuticle. Indeed, this latter was wanting over a space as large as a shilling, where an ulcer existed. The head was large, measuring 19 inches in circumference. The right foot was affected with talipes varus. The movements of the members were feeble. The urine ran away *guttatim*.

*Oct. 9th.* I injected the tumor in the usual manner, using 1-16 grain of iodine with twice that quantity of iodide potassium in half a drachm of water. Some reaction followed, which soon subsided; and, at the end of a week, the tumor could be kept at a level with the sound skin by the applicatian of collodion. This was continued, and the sac did not fill again.

The child lived seven months after this operation, and died of hydrocephalus, for which it received treatment, which I have detailed in a separate article on that subject.

Case V.—A female child, aged four months, was brought to me, March 11th, 1855, with a spina bifida, situated at the middle of the lumbar division of the spinal column. The tumor was two and a half inches in length, one and a half in breadth, and about an inch in elevation. It presented the usual symptoms, except that an ulcerated surface of the size of a quarter of a dollar existed at the centre. The fluid could be, in a great measure, pressed into the spinal column, the child becoming uneasy. The head was a little larger than natural, and the sutures at the superior part not closed. In other respects, the child was well formed and healthy.

*March 12th*, 1855. Injected the tumor with one drachm of a solution containing one-eighth of a grain of iodine, three-fourths of a grain of iodide potassium, to one drachm of distilled water. I was assisted at this time by Prof. Freer, and Drs. Fisher and Morfit, of this city. On other occasions, Dr. T. Rush Spencer and several physicians visited the case with me.

This injection was followed by some heat and fullness, which subsided, and the tumor became more flaccid. Light compression was then used upon it.

*March 17th*. Repeated the same injection; also, March 22, 25, 28, and April 1, 10 and 14. At the end of this time the surface of the tumor was on a level with the sound skin, the ulceration was healed, and the fluctuation had entirely disappeared. Before each injection, a quantity of fluid equal to that of the solution to be thrown in was withdrawn.

This child was delicate, and the symptoms of a febrile nature after each operation were greater than I have before witnessed in any case. This I attributed to impurity of the iodide employed, and after changing it, less effect was noticed. At first, the fluid withdrawn was limpid, but after each injection it became turbid, of a whitish hue, and had flakes of lymph and albumen floating in it.

The child returned home, April 20, with every appearance of being cured, and with the promise on the part of the parents that should the symptoms of hydrocephalus increase, they would return to have the head injected. This they did not do; and in June, 1856, fourteen months after the completion of the treatment, I learned authentically that the child was in good health, the spina bifida perfectly cured, and the head not materially worse than at the time of the treatment being employed.

CASE VI.—This was a child, aged three months, in good health, except a tumor of the size of half an orange, situated at the lower part of the lumbar region, with all the characteristics of spina bifida. The case was intrusted to the care of Dr. S. G. Crawford, whom I furnished with the instrument I was in the habit of using.

Dr. Crawford used the injection three times, at intervals of two weeks, applying the collodion when it was soft, at the end of which time the tumor was level with the surrounding skin. A pan, supported by a band, was then applied.

The third injection in this case was composed of four grains of iodine and ten grains of iodide of potassium to the ounce of distilled water. Of this two drachms were thrown in. The first two were of half the above strength.

Six months afterward, the case was reported perfectly cured.

CASE VII.—This case occurred in the practice of Dr. Crawford while he was treating the last one.* It was in a new-born infant. The tumor was situated near the end of the sacrum and was pediculated; it was of the size of a hen's egg, the integuments thick, and the fluctuation obscure. The child was perfectly well formed in other respects.

In view of the slight effects produced by the injection in the former case, Dr. Crawford resorted at once to a solution con-

* The year 1848 was the first of a series of years in which the cholera prevailed. The terror produced by the disease caused many malformations, besides changing the usual relations between the numbers of male and female births, as has been noticed elsewhere.

taining iodine one grain, iodide potassium three grains, to one drachm of distilled water. After drawing off considerable fluid, this was injected, allowed to remain about a minute, and then pressed out in part. A compress and roller were then applied over it.

The inflammation which resulted was acute, but gave rise to no cerebral or spinal symptoms, and subsided at the end of five days. Afterwards, the compression was increased and continued for a month. At the end of this time, the tumor was hard, wrinkled and without fluctuation, about one-fourth of its original size.

Four months afterward, it remained in the same state and seemed perfectly cured.

CASES OPERATED IN PARIS.

M. Chassaignac performed the operation in June, 1851. He injected equal parts of tincture of iodine and water, first evacuating the tumor and placing the finger so as to prevent the injection from passing into the spinal opening. After a short sojourn, the solution was allowed to flow off.

For six weeks there was no change in the tumor, but at the end of that time it disappeared.

M. Debout stated that he had very recently seen M. Velpeau practice the same operation. No ill effect resulted. I am not aware that M. Velpeau has published his observations.

The case of M. Nelaton is reported in the *Gazette Medicale* for June 4, 1854. The subject was a new-born child; the tumor was pediculated. M. Nelaton drew off the water and injected tinct. of iodine, which was retained a few minutes and then evacuated. The pedicle of the tumor was compressed during the operation, and afterwards a gum elastic ligature was placed lightly around the base. At the end of fifteen days no change had been noticed, except a little increased firmness. No dangerous symptoms occurred.

The progress of physiology enables us to understand and explain some of the circumstances connected with spina bifida which have heretofore been considered as contra-indicating any operation.

*A. The paralysis and contraction of the lower extremities.*

That disease or imperfect development of the spinal cord at one point would produce paralysis of the lower extremities, is a fact which has long been known; but that the same lesions might give rise to permanent contraction of the muscles of those members, is a fact more recently established. Marshall Hall, Brown-Sequard and others, have proved this by their experiments on animals. Surgeons have witnessed it as the effect of injuries. In a man of fifty-five years of age, who received an injury of the spine at the top of the dorsal division, producing at first perfect paralysis, contraction of the lower members took place, flexing the thigh upon the pelvis, and the legs upon the thigh, firmly for over two years, at the end of which time the patient died. "Separation of the muscles from the brain increases their irritability" (Marshall Hall, *Med. Chir. Review*, vol. 22).

It is true that Marshall Hall was also of the opinion that separation of the muscles from the spinal cord destroys their irritability. There are two facts in regard to the pathological anatomy of spina bifida which bear upon this point:

1st. Ruysch has remarked that the attenuation of the cord rarely extends below the opening in the spine.

2nd. In cases of spina bifida of the sacrum or lumbar region, the spinal cord is longer than natural. "It is prolonged to the end of the sacrum" (*Ollivier d'Anger*, *Traite des Maladies, de la Moelle Epiniere*).

We see, therefore, that the cases in which the muscles have no connection with the brain must be most frequent. When the tumor is situated at the extremity of the cord only should we expect paralysis without contraction. It is to be regretted that in but few reports of cases of this malformation have the appearances on dissection and the symptoms been described with sufficient accuracy to enable us to judge of their relations to each other.

An operation, if successful, is often followed by cure of the paralysis and the deformity from muscular contraction. This effect was witnessed in the first case operated by me, already reported, in the case reported by Dr. Latil de Thimecour (*Gazette Medicale* for 1845, p. 764, *et. seq.*) the same thing occurred, and also in the two cases cited by Messrs. Probart and Rosetti.

Experiments on animals and observation on man have abundantly shown that extensive injuries of the brain, spinal cord and nerves are capable of being repaired. Thus, M. Flourens has found that nerves could be crossed and unite the extremity of one to the root of another, and *vice versa.* Brown-Sequard has found that when a piece of the cord is removed in some animals, it is regenerated. Dessault reports the case of a soldier in whom the cord was divided, and who recovered and retained the power of voluntary movements. Flourens also found that extensive longitudinal wounds of the cord in the goose were healed so as to leave no paralysis behind.

*B.* The spasms, so frequent and so fatal in spina bifida, are not owing to any incurable, organic, or functional disease of the nervous system, but to want of the natural pressure on the central organs, the walls of the tumor not giving sufficient support to the fluid within.

That want of pressure on the brain gives rise to spasms and insensibility, is a fact which has been often observed, and which I have particularly insisted upon in the report of a case of hydrocephalus. But it does not appear to have been so generally admitted as it deserves to be; I therefore add here the testimony of others. And I am particularly gratified to be able to quote so just and competent an observer as M. le docteur, Jules Guerin. He says (*Gazette Medicale* for 1842, p. 202): "If too much pressure on the tumor produces paralysis always, and loss of consciousness often, it is beyond dispute that the sudden cessation of the compression which the contents of the sac exercise on the cord, may produce the same accidents. We know the experiments of Magendie on the effects of abstracting the cerebro-spinal liquid. We recall the interesting observation of M. A. Berard, who, after having trephined a patient fourteen times for a fungus of the dura mater, saw his patient fall senseless when the brain was uncovered, and could only restore him by using strong compression of its substance. The unfavorable influence of this want of compression is shown more directly and positively in the second case of M. Dubourg, in which the little patient, which cried strongly at the commencement of the operation, lost all movement, and remained senseless when the tumor was evacuated. We remember also, in 1836, accompanying M. Philip Boyer to see an infant treated by repeated punctures. We several times saw the patient lose all consciousness when too much water was evacuated, and could only be rendered conscious by a tight bandage and compress upon the tumor."

These effects do not differ much from those observed in animals dying of hemorrhage, in which syncope and convulsions are observed.

As the presence of the cerebro-spinal liquid antagonizes the pressure of the blood, the sudden removal of the former may either induce a syncope, or, if this be avoided, a fullness of the vessels of the brain, as has been stated by Todd and Bowman.

If an operation is successful, although a bony arch may not be formed, yet the opening of the spine is closed up by a dense, firm tissue, which supplies its place.

### APPLICATION OF THE DIFFERENT OPERATIONS PROPOSED.

1. We may, I think, pass by as unworthy of consideration the treatment by the seton, caustic and pressure, as having never produced a radical cure in a single instance, except the latter, perhaps. It is doubtful if the case reported by Sir Astley Cooper, in which a child retained the fluid in the spine by means of a truss, is to be regarded as in any degree palliated. Certain it is, that pressure is generally not only inapplicable, but dangerous if used to a great degree.

2. Excision will probably never be resorted to by any one who has carefully studied the subject. The objections to it are the liability to divide the nerves, and even the cord itself, when adherent to the walls of the tumor; the sudden removal of the liquid, and the entrance of air into the opening, both of which are attended with danger; the great difficulty in securing union by the first intention throughout the whole extent of the wound; finally, because, in the express language of Vidal, "all those who have tried it have found it to result fatally" (*Traite de Pathologie Externe*, vol. 5, p. 765).

3. *Puncture.*—There are many cases reported in which it is difficult to determine from the description whether tapping or acapuncture was performed. In reference to puncture, whether with a lancet or trochar, the same remarks apply to this operation which we have made concerning excision. The name and authority of Cooper have been sufficient to give it currency; but a careful examination of the cases in which he used it will lead to a conclusion not favorable to its repetition. In one case, Sir A. Cooper used a sharp needle. After repeated attacks of spasm and inflammation, the patient remained without a cure, although the accidents did not prove fatal. The cases of Sir Philip Crampton, of Stevens and several others, were by acapuncture, which is free from many of the objections of the former.

With the exception of such cases, the operation has uniformly failed. Brechet, Pacoud and Bajard have used it several times each, and have never found it to succeed. Chelius says that repeated punctures nearly always fail. The reason for this difference is obvious. The punctures produce an inflammation of the walls of the tumor by which they are thickened, with less danger of ulceration than results from larger wounds. The evacuation of the liquid has no tendency to cure the disease, and is not free from danger, as I have shown.

4. The ligature, proposed by Forestius and recommended by B. Bell, has recently been revived by Reynard and L. de Thimecour. The former, by placing a large quill on each side of the root of the tumor, and passing the ligature through the hollow of each, tying it so as to strangulate it; the latter using, instead of the quills, two rods of wood, but placing them in the same position so as to act in the same way. I confess that if it were not for the superior claims of the injections of iodine, this is the method which I should prefer among all those at present known. It has the advantage of not evacuating the fluid suddenly, of not exposing the membranes to the contact of air, and if excision be allowable under any circumstances, it must be in connection with compression, as has several times been done already. There is a drawback upon the value of this method, which is, that in the greater number of cases, according to my observations, the tumor is so flat as not to admit of such an application being made, even. This is particularly the case in new-born infants; the cases of large and pediculated tumors being those mostly of patients of more advanced age.

Thus far its application has been followed by more favorable results than the use of either of the preceding methods of treatment.

5. *Injections of Iodine.*—This method of treating spina bifida is said to have been first proposed by Velpeau. The only passage of his writings which I am am acquainted with bearing upon this point is the following:

"It will be readily understood that it is not the *manuel operatoire* which embarrasses, it is the danger of the operation itself; but how great the object in attacking spina bifida, hydrocephalus, etc., by the aid of a remedy, an operation, which succeeds in so many cases, has so few inconveniences when applied to other cavities of the same nature."

"On the other hand, how shall we go to work? *Who will dare the first to throw the tincture of iodine into a spina bifida, or into the cavity of the cranium, knowing that inflammation of the meninges is rapidly mortal?*"*

In his "Essay on Medicated Injections," published in 1846, M. Velpeau alludes to the subject, but does not recommend these operations (see p. 123).

This notice from such a source certainly is not well calculated to encourage a trial. I think, therefore, it is not too much to claim for myself whatever of merit there may be in recommending and carrying into execution the injection of iodine into the cerebro-spinal cavity. This point, besides, is not, that I know, by any means contested in France. M. Monod, in a report made to the Society of Surgery, at its sitting of the 16th March, 1854, says: "M. Brainard had the honor of presenting, in 1847, the first case of cure of spina bifida, by iodine injections, known to science."

* Recherches sur les Cavites Closes, 1843, p. 191.

Although the value of any method of treatment must be determined by its results, yet the conformity of its mode of action to natural processes may weigh much in its favor. What is desired in cases of spina bifida is a means of inducing, with certainty, upon the internal surface of the sac of the tumor, a deposit of lymph, and in the walls of the tumor an adhesive inflammation of a moderate degree of intensity, without wound of the covering, which is often quite abnormal in structure.

It seems obvious to me that injections of iodine in solution, judging *a priori*, are more likely to accomplish these objects than any of the modes of treatment hitherto employed. There may indeed be different opinions about the best method of using it, but experience will reconcile these. MM. Chassaignac, Velpeau and Nelaton have preferred to evacuate the tumor, and after injecting it with tincture of iodine, letting this also flow out. The rules which I have adopted are as follows:

1. Make the puncture in the sound skin at the *side* of the tumor.

2. Inject the solution of iodine and iodide of potassium, commencing with $\frac{1}{4}$ grain of the former to $\frac{3}{4}$ of the latter, and retain it by slight pressure.

3. Evacuate no more of the liquid than the quantity of injection about to be thrown in.

4. If convulsions supervene, the fluid may be drawn out and its place filled with distilled water. (I have not had occasion to resort to this practice more than once.)

5. Lay the patient on the face, and if there be heat, apply evaporating lotions to the tumor and to the head.

6. When the tumor becomes flaccid, apply collodion or other means of contracting the skin. This should be continued for some months after the swelling has disappeared.

7. After the effect of an injection is past, repeat it as many times as may be necessary, and gradually increase the strength according to the effect produced.

By using these precautions, I have thus far avoided serious accidents, although four out of five of the cases which I have had occasion to treat were affected at the same time with chronic hydrocephalus. In new-born infants, I should be disposed, until experience shall have further enlightened us, to adhere to this practice. In older persons, and particularly when the tumor is pediculated, I should not hesitate to follow the practice adopted by the French surgeons. Theoretically considered, it would appear that the solution injected ought to be applied to the surface which secretes the fluid, which, as we have seen, is probably the pia mater; but in practice it seems to have been sufficient to thicken the covering of the tumor to effect a cure of the disease.

### CASES IN WHICH TREATMENT IS INAPPLICABLE.

In advising an operation for the cure of spina bifida, I would by no means be understood as recommending its application without regard to the peculiarities of each case. I do not, however, regard any rules heretofore given as being in all respects sufficient on this point.

The following are the circumstances in which I have advised against interference:

1. When the tumor is small, and the patient several years of age and in good health.

A young lady, eighteen years of age, well formed and in good health, except a spina bifida at the junction of the cervical and dorsal regions, was brought to me for advice. The tumor had existed from birth, but was not increasing in size; was tense during sleep, and flaccid at other times; could be, in a great measure, pressed into spine, but much pressure caused uneasiness. This girl was in good health, excepting that the mother stated her to be nervous, and subject to starts at slight noises and unexpected sights. I advised against interference.

2. When the child is laboring under acute disease, whether this be of the head, of the spinal cord, convulsions, etc.

I do not consider paralysis and contraction of the lower members a contra-indication. They depend upon a state of the cord which may be cured, and they have, in several instances, disappeared after a cure, as before stated.

Neither is hydrocephalus or double tumor to be regarded as a bar to the operation, although, no doubt, they add to the danger of the disease. Four out of five of the cases I have treated had hydrocephalus, but in no instance did the treatment fail to cure the spina bifida. The only doubt I should have in this case would be whether the head or back should be first treated. (This part of the subject I have noticed in an article on Hydrocephalus.)

Still less are the existence of umbilical hernia, unless of unusual size, ulceration on the surface of the tumor or its great vertical extent, to be regarded as serious objections. Two of the cases treated by myself had ulceration on the tumor, one had cicatrices, obviously the result of ulceration, but all these were cured.

It would only be a combination of these complications very unusual, or evidence of serious malformation of the nervous centres, which would induce me to abandon the case as incurable.

Of the abnormal states of the cord noticed by Meckel as associated, viz., enlargement, flattening, division into two parts, it is doubtful if either is an obstacle to a use of the members in case the disease is cured.

NOTE.—M. Paget, in 1854, treated a tumor of large size by subcutaneous ligature. "A strong, double silk ligature was passed subcutaneously around the tumor, the ends being finally brought out in the middle of its upper part." The ends were tied loosely and attached to a band of elastic webbing, which was kept tense. For three days no effect was produced; the child died on the eighth day. The vessels of the spinal cord were found much injected; no other appearance of inflammation (*North-American Med. Chir. Review* for Nov., 1858, p. 1117; from *Med. Times*).

The spinal tumor sometimes communicates with the ventricles through the canal in the centre of the cord. A case where this occurred is related in the Transactions of the Pathological Society of London (ib., p. 1120).

When this is the case, the tumor sometimes diminishes when the head increases, or the reverse.

A case of spontaneous cure, and another where it seemed to be cured by an ulcer on the sac, are related in the *Med. Chir. Review* for Nov., 1858, p. 1120.

